# Prognostic Nutritional Index Considering Resection Range Is Useful for Predicting Postoperative Morbidity of Hepatectomy

**DOI:** 10.1007/s11605-020-04893-z

**Published:** 2021-01-08

**Authors:** Shigeyuki Nagata, Shohei Maeda, Satoko Nagamatsu, Seiichiro Kai, Yasuro Fukuyama, Seigo Korematsu, Hiroyuki Orita, Hideaki Anai, Hiroyuki Kuwano, Daisuke Korenaga

**Affiliations:** 1 Department of Surgery, Nakatsu Municipal Hospital, 173 Shimoikenaga, Nakatsu, Oita 871-8511 Japan; 2 Department of Pediatrics, Nakatsu Municipal Hospital, Oita, Japan; 3 Department of Surgery, Oita Medical Center, Oita, Japan; 4 Department of Surgery, Fukuoka City Hospital, Fukuoka, Japan

**Keywords:** Prognostic nutritional index, PNI, Hepatectomy, Morbidity, Hepatocellular carcinoma

## Abstract

**Background:**

Poor preoperative nutritional and immunological status are major risk factors for postoperative complications in patients with various malignancies. Lower preoperative prognostic nutrition index (PNI) is associated with higher rates of postoperative complications and poorer prognosis in those patients. The aim of this study was to analyze the predictive value of the PNI for post-hepatectomy complications in patients with hepatocellular carcinoma (HCC), and evaluate its utility in the surgical procedure.

**Methods:**

This retrospective study included 510 patients who underwent open hepatectomies for HCC. The predictive value of the preoperative nutritional and immunological status for postoperative complications was assessed using the PNI. Postoperative complications were defined as grade II or higher per the Clavien-Dindo classification. Postoperative complication rates were compared according to surgical procedure (major hepatectomy vs minor hepatectomy).

**Results:**

Patients with postoperative complications had significantly lower PNIs than those without (43.1 ± 5.5 vs 47.0 ± 5.7, *P* < 0.001). In the multivariate analysis, low preoperative PNI (< 45) was an independent risk factor for postoperative complications after hepatectomy (hazard ratio, 3.85). When patients were classified per their PNI (high vs low) and extent of surgical procedures (major vs minor), there were more complications among patients with low PNI than those with high PNI, regardless of the extent of surgical procedures. Specifically, the group of patients with low PNI who underwent major hepatectomy had significantly higher rates of postoperative complications than the other groups**.**

**Conclusions:**

Adding the resection range to the PNI is useful for predicting the postoperative morbidities of hepatectomy patients.

## Introduction

Hepatocellular carcinoma (HCC) is one of the most common malignancies worldwide.^1^ The main curative treatment for HCC is hepatic resection. In the past several decades, advances in surgical techniques, optional treatments, and perioperative care have significantly improved postoperative outcomes.^2^–^5^ However, the postoperative morbidity rate remains high. Postoperative complications have a substantial negative impact on the postoperative course and oncological outcomes.^6^–^9^

Previous studies have identified several risk factors for postoperative morbidity after hepatectomy for patients with HCC.^10^–^15^ Preoperative poor nutritional and immunological status have been associated with postoperative morbidity and poor long-term outcomes of patients with malignant tumors.^16^–^21^ The prognostic nutritional index (PNI) is a widely used combined measure of the nutritional and immunological status, and is calculated using serum albumin concentrations and total lymphocyte counts in the peripheral blood. Initially, it was proposed as a marker for predicting the prognoses of patients with gastrointestinal malignancies.^22^ Clinical studies have suggested that the preoperative PNI is associated with postoperative complications and the prognoses of patients with several digestive malignancies.^23^–^27^

A few studies have demonstrated the predictive significance of the PNI in patients with HCC undergoing hepatectomy.^28^–^30^ However, in these, different surgical procedures were selected based on the sizes or locations of the HCC tumors, and the relationship of the PNI to the extent of hepatic resection has not been described to date. In the present study, we retrospectively analyzed the predictive value of the PNI for post-hepatectomy complications in patients with HCC and assessed its utility in the surgical procedure**.**

## Methods

### Patients

A total of 510 patients with HCC who underwent open hepatectomies between January 2003 and December 2016 at the Fukuoka City Hospital, the Oita Medical Center, and the Nakatsu Municipal Hospital were included in this study. Patients who underwent laparoscopic surgery were excluded. Our study was approved by the Institutional Ethics Committees of the hospitals involved, and participants were allowed to opt out (approval number: NMH2019034).

### Investigational Variables

Preoperative blood samples were obtained from patients within 2 weeks prior to undergoing surgery. The preoperative factors evaluated as potential predictors of postoperative morbidity in patients included patient age, sex, and body mass index; presence of the surface antigen of hepatitis B virus (HBV) or antibody to hepatitis C virus (HCV); diabetes mellitus; the American Society of Anesthesiologists (ASA) physical status class; primary or recurrent HCC; serum concentration of albumin, total bilirubin, total lymphocyte count of the peripheral blood, and prothrombin time; indocyanine green retention rate at 15 min (ICGR15); the Child-Pugh classification; and serum concentrations of alpha-fetoprotein (AFP) and des-γ-carboxy prothrombin (DCP). The PNI was calculated as follows:

$$ \mathrm{PNI}=10\times \mathrm{albumin}\ \mathrm{g}/\mathrm{dL}+0.005\times \mathrm{lymphocyte}\ \mathrm{count}/{\mathrm{mm}}^3 $$

Patients with grade II or higher complications according to the Clavien-Dindo classification (CDC) were defined as experiencing postoperative complications. Data were collected from patients’ records.

To analyze the occurrence rates of postoperative complications due to the surgical procedures, information on the resection range was collected from the surgical records.

### Definitions and Surgical Procedures

Major hepatectomy was defined as the following: (1) resection of 3 or more contiguous liver segments according to Couinaud’s classification or (2) resection of the right posterior, and anterior resection because of the unique and advanced techniques required. Minor hepatectomy included partial resection and segmentectomy except the above.

The details of the surgical techniques and patient selection criteria have been described.^31^ Criteria for hepatic resection included the presence of ascites that was either undetectable or controlled with diuretics; serum total bilirubin concentration less than 2.0 mg/mL; and ICGR15 less than 40%. Parenchymal transection was carried out using an ultrasonic dissector with a coagulator. All sizable vessels were ligated along the transection line. Inflow vascular control was carried out with intermittent hemi- or total Glisson’s sheath occlusion (Pringle maneuver). Inflow occlusion was applied intermittently with 15 min of occlusion alternating with 5 min of reperfusion.

### Statistical Analyses

We performed a receiver operating characteristic (ROC) curve analysis of postoperative complications to evaluate the ability of the optimal cutoff values of the preoperative total bilirubin, albumin, lymphocyte count, prothrombin time, ICGR15, and PNI of interest to predict postoperative outcomes. Goodness of fit was assessed by calculating the area under the curve (AUC), and the optimal cutoff values were determined using Youden’s index.

Chi-square tests and Mann-Whitney tests were used to compare preoperative factors and surgical factors between the two groups (complication group vs no complication group). Univariate and multivariable analyses were performed using a logistic regression model. To identify potential predictors of postoperative complications, several preoperative variables that were found to be independent in the univariate analysis were included in the multivariable analysis. All statistical analyses were performed with EZR (Saitama Medical Center, Jichi Medical University, Saitama, Japan), which is a graphical user interface for R (The R Foundation for Statistical Computing, Vienna, Austria). This is a modified version of R commander, which is designed to add statistical functions that are frequently used in biostatistics (http://www.nature.com/bmt/journal/v48/n3/pdf/bmt2012244a.pd). *P* < 0.05 was considered statistically significant.

## Results

### Postoperative Complications

Postoperative complications occurred in 132 of 510 patients (25.9%). Of these, 34 (6.7%) presented with critical complications (CDC grade III or higher), including bile leakage (*n* = 10, 1.9%) and hepatic failure (*n* = 9, 1.8%), as shown in Table 1. Of the total 510 patients, 15 died while admitted (2.9%). The patients who underwent hepatectomy for HCC were divided into two groups based on the presence or absence of complications (no complication and complication groups, respectively), as shown in Table 2. Compared with the no complication group, the complication group had a higher rate of HCV positivity (54.2% vs 66.7%, *P* = 0.013), an ASA class III (16% vs 37%, *P* < 0.001), and a Child-Pugh class B (5.8% vs 12.1%, *P* = 0.021). Furthermore, the levels of preoperative total bilirubin (0.99 vs 0.85, *P* = 0.013) and ICGR15 (18.7 vs 16.2, *P* = 0.023) were higher in the complication group than in the no complication group. The levels of preoperative albumin (3.62 vs 3.93, *P* < 0.001), lymphocyte counts (1385.4 vs 1552.7, *P* = 0.003), prothrombin time, and PNI (43.1 vs 47.0, *P* < 0.001) were also significantly lower in the complication group than in the no complication group. Deceased patients (CDC grade V) had significantly lower PNI than those in the no complication group (44.3 vs 47.0, *p* = 0.031).

**Table 1:** Critical complications (Clavien-Dindo classification III–V)

Postoperative complication	*n* (death)
Cardiac complication	3 (1)
Sepsis	2 (2)
Postoperative hepatic failure	9 (6)
Postoperative bleeding	2 (1)
Bile leakage	10 (2)
Pneumonia	2 (2)
Intraoperative excessive bleeding	1 (1)
Portal venous thrombosis	1
Intra-abdominal abscess	1
Dehiscence	1
Unknown	2

**Table 2:** Patient characteristics between no complication and complication groups

Variables	No complication (*n* = 378)	Complications (*n* = 132)	*P* value
Age (years)*	67.3 (61, 75)	68.3 (62, 74)	0.279
Sex (male/female)	290/88	96/36	0.409
Body mass index (kg/m^2^)*	23.5 (21.1, 23.3)	23.1 (20.9, 23.0)	0.239
Etiology (HBV/HCV)	98/205	24/88	0.013
Diabetes mellitus	112 (29%)	44 (33%)	0.442
ASA-PS class III	61 (16%)	49 (37%)	< 0.001
Recurrent HCC	87 (23%)	29 (22%)	0.809
Preoperative albumin (g/dL)*	3.93 (3.7, 4.2)	3.62 (3.3, 4.0)	< 0.001
Preoperative total bilirubin (mg/dL)*	0.85 (0.60, 0.99)	0.99 (0.65, 1.18)	0.013
Preoperative lymphocyte count (/μL)*	1552.7 (1138, 1479)	1385.4 (929, 1309)	0.003
Preoperative prothrombin time (s)*	91.2 (83.6, 100.0)	87.6 (79.5, 95.2)	0.013
ICGR15 (%)*	16.2 (9.0, 21.0)	18.7 (12.0, 25.0)	0.023
Child-Pugh class (A/B)	356/22	116/16	0.021
AFP (ng/mL)*	709.6 (4.8, 38.8)	1274.6 (8.2, 138.5)	0.560
DCP (mAU/L)*	2252.8 (19.0, 506.5)	2089.8 (19.0, 373.0)	0.651
PNI*	47.0 (43.5, 47.3)	43.1 (39.5, 42.5)	< 0.001

*HBV*, hepatitis B virus; *HCV*, hepatitis C virus; *ASA-PS*, American Society of Anesthesiologists physical status; *HCC*, hepatocellular carcinoma; *ICGR15*, indocyanine green retention rate at 15 min; *AFP*, alpha-fetoprotein; *DCP*, des-γ-carboxy prothrombin; *PNI*, prognostic nutritional index

*Mean (25th percentile, 75th percentile)

### Predictors for Postoperative Morbidity

**Fig. 1:**
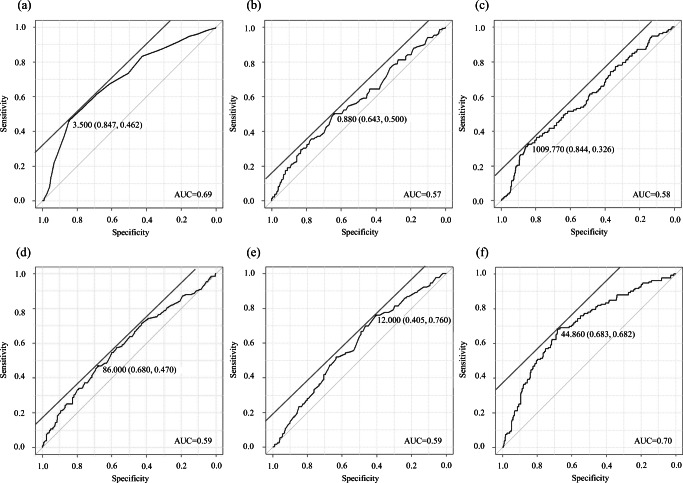

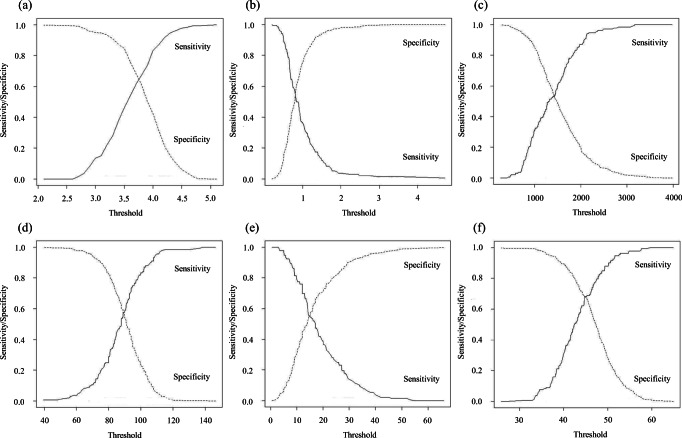
Cutoff points were determined by analyzing the receiver operating characteristic (ROC) curve for the total albumin level (**a**), total bilirubin level (**b**), lymphocyte count (**c**), prothrombin time (**d**), indocyanine green retention rate at 15 min (ICGR15) (**e**), and prognostic nutritional index (PNI) (**f**)

To evaluate the preoperative risk factors against postoperative complications, cutoff points for total bilirubin and albumin levels, lymphocyte count, prothrombin time, ICGR15, and PNI were determined by analyzing the ROC curve (Fig. 1). The optimal cutoff for the PNI was 44.8 (sensitivity and specificity of 0.682 and 0.683, respectively). Of the total 510 patients, 214 (42%) had preoperative PNI that were lesser than 45 (low-PNI group), and 296 (58%) had PNI of 45 or greater (high-PNI group). The morbidity rate was significantly higher in the low-PNI group than in the high-PNI group (42.1% vs 14.2%, *P* < 0.0001). A similar trend was observed for the rate of CDC grade III or higher complications (10.3% vs 3.7%, *P* = 0.0035). Postoperative stays were significantly longer in the low-PNI group than in the high-PNI group (25.4 days vs 19.6 days, *P* < 0.0001). Univariate analyses of preoperative factors were performed to predict postoperative complications. Factors significantly prognostic for postoperative complications are shown in Table 3. Multivariable analysis identified two factors that were prognostic of morbidity after hepatectomy in patients with HCC (ASA-PS class III and PNI < 45), as shown in Table 3. Preoperative PNI less than 45 was a more powerful independent predictor of postoperative complications (hazard ratio, 3.85; 95% confidence interval, 2.50–5.94; *P* < 0.0001) (Table 3).

**Table 3:** Univariate and multivariate analyses of preoperative risk factors associating postoperative complication after hepatectomy for HCC

Variables	Univariate analysis		Multivariate analysis	
	HR (95% CI)	*P* value	HR (95% CI)	*P* value
ASA-PS class III	3.06 (1.91–4.90)	< 0.0001	2.38 (1.48–3.81)	< 0.0001
Albumin < 3.6 g/dL	3.82 (2.47–5.94)	< 0.0001		
Total bilirubin >0.9 mg/dL	1.83 (1.20–2.80)	0.0034		
Lymphocyte count < 1000/μL	2.53 (1.54–4.13)	0.0001		
Prothrombin time < 86 s	1.87 (1.22–2.87)	0.0028		
ICGR15 > 12%	2.07 (1.28–3.39)	0.0016		
Child-Pugh class B	2.22 (1.05–4.61)	0.0216		
PNI < 45	4.36 (2.80–6.86)	< 0.0001	3.85 (2.50–5.94)	< 0.0001

*HR*, hazard ratio; *CI*, confidence interval; *ASA-PS*, American Society of Anesthesiologists physical status; *ICGR15*, indocyanine green retention rate at 15 min; *PNI*, prognostic nutritional index

### Analysis in Classified Surgical Procedure

**Fig. 2:**
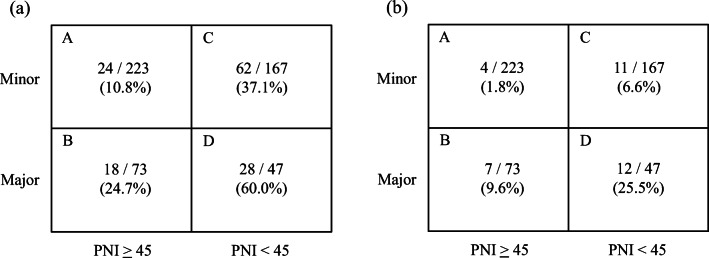
The total 510 patients were divided into four groups according to their prognostic nutritional indices (PNIs) and surgical procedures. **a** The total numbers of patients in each group and patients with CDC grade II or higher complications are as follows: 24/223 (10.8%) in column A, 18/73 (24.7%) in column B, 62/167 (37.1%) in column C, and 28/47 (60.0%) in column D. The values in parentheses indicate rates of complications. High-PNI patients undergoing minor hepatectomy (A) and major hepatectomy (B). Low-PNI patients undergoing minor hepatectomy (C) and major hepatectomy (D). A vs B, *P* < 0.001; C vs D, *P* = 0.011; A vs C, *P* < 0.001; B vs D, *P* = 0.017; A vs D, *P* < 0.001; B vs C, *P* = 0.059. **b** The total numbers of patients in each group and patients with CDC grade III or higher complications are as follows: 4/223 (1.8%) in column A, 7/73 (9.6%) in column B, 11/167 (6.6%) in column C, and 12/47 (25.5%) in column D. A vs B, *P* = 0.002; C vs D, *P* < 0.001; A vs C, *P* = 0.015; B vs D, *P* = 0.019; A vs D, *P* < 0.001; B vs C, *P* = 0.416

Results of the analysis of operative factors in the no complication and complication groups are shown in Table 4. Minor hepatectomies were more frequent in the no complication group (80.4%) compared to the complication group (65.5%). The complication group had longer operative time (303 min vs 267 min, *P* = 0.001), more blood loss (1778 g vs 774 g, *P* < 0.001), higher rate of intraoperative blood transfusion (41.7% vs 14.8%, *P* < 0.001), and longer postoperative hospitalization (33.2 days vs 18.1 days, *P* < 0.001) compared with the no complication group. In patients who underwent major hepatectomy, the surgical times were not significantly different between the high- and low-PNI groups (405.7 min vs 427.3 min, *P* = 0.226); however, significant differences were noted in the blood losses (1598.2 mL vs 3115.9 mL, *P* = 0.030) and duration of postoperative hospitalization (26.8 days vs 39.4 days, *P* = 0.013). Preoperative PNI did not differ significantly among surgical procedures (minor hepatectomy vs major hepatectomy groups; 45.7 vs 46.6, *P* = 0.091). Morbidity rates were significantly higher in the major hepatectomy groups (38.3%) than in the minor hepatectomy groups (22.1%). As shown in Fig. 2a, patients were divided into four groups according to the PNI and surgical procedures. Patients with low PNI presented with significantly higher rates of postoperative complications than did the patients with high PNI (low PNI vs high PNI in the minor hepatectomy group, *P* < 0.001; low PNI vs high PNI in major hepatectomy group, *P* < 0.001). Specifically, patients with low PNI who underwent major hepatectomy presented with significantly higher rates of postoperative complications than those of other groups (high PNI in minor hepatectomy group vs low PNI in the major hepatectomy group, *P* < 0.001; minor vs major hepatectomy in the low-PNI group, *P* = 0.007). There were no differences between patients with high PNI who underwent major hepatectomy and patients with low PNI who underwent minor hepatectomy (*P* = 0.074). A similar trend was observed for the rate of CDC grade III or higher complications (Fig. 2b).

**Table 4:** Surgical outcome in 510 patients who underwent hepatectomy for HCC

Variables	No complication (*n* = 378)	Complications (*n* = 132)	*P* value
Resection type			
Major hepatectomy	74	46	< 0.001
Minor hepatectomy	304	86	< 0.001
Operation time (min)*	267 (195, 324)	303 (219, 355)	0.001
Blood loss (g)*	774 (290, 1035)	1778 (490, 2076)	< 0.001
Blood transfusion rate (%)	14.8	41.7	< 0.001
Postoperative hospital stay (days)*	18.1 (14, 20)	33.2 (18, 40)	< 0.001

*HCC*, hepatocellular carcinoma

*Mean (25th percentile, 75th percentile)

## Discussion

PNI is one of the few nutritional assessment tools listed in the guidelines of the Japanese Society for Parenteral and Enteral Nutrition (JSPEN) to assist patients with treatment selection and to predict prognosis. In this study, we investigated the relationship between preoperative PNI and surgical outcomes after hepatic resection for patients with HCC. Multivariate analysis revealed that PNI was an independent predictor of postoperative complications in patients with HCC who underwent hepatic resection. The PNI is a simple and effective parameter and was initially created to evaluate preoperative nutritional and immunological conditions.^27^ The PNI is derived from two parameters, serum concentration of albumin and total lymphocyte count. Serum albumin level is a fundamental nutritional assessment indicator, but albumin alone is no longer considered a marker of nutritional status.^32^ It is a negative acute-phase reactant and, in the presence of liver disease, acts as an indicator of decreased hepatic synthetic function. Therefore, it is difficult to assess the nutritional status of cirrhotic patients, who comprise 70–90% of patients with HCC. It has been reported that preoperative serum albumin was associated with operative morbidity and mortality after surgery, including hepatectomy.^16^,^17^,^33^ Previous studies have suggested that lymphopenia and some tools combined with the peripheral lymphocyte count were associated with the prognosis of patients with malignant tumors.^18^,^20^,^21^ Therefore, we hypothesized that the PNI, which combines these parameters, would be a useful predictor of prognosis for patients with HCC.

Other tools to assess nutritional status may be used, such as the Nutrition Risk Screening 2002 (NRS 2002)^34^ and the recently published Global Leadership Initiative in Malnutrition (GLIM) criteria.^35^ These tools are excellent for the diagnosis of malnutrition, but they include several factors and are time-consuming for diagnostic purposes. The most beneficial aspect of the PNI is its easy calculation and rapid determination by blood exam data, which are routinely collected for surgical patients in the hospital. Therefore, the PNI is suitable as an initial assessment tool.

The PNI was initially used in Japan to assess the immunologic and nutritional aspects of patients with various malignancies who underwent surgical treatments.^22^–^26^ These studies proposed that the incidence of postoperative complications was more frequent in patients with low PNIs than in those with high PNIs. In several studies, as in ours, the cutoff value of the PNI is approximately 45.^22^,^24^,^29^,^30^ In patients with HCC, several researchers have demonstrated that the PNI is a predictive indicator of morbidity or mortality after hepatectomy,^28^–^30^ but this remains controversial.

In the present study, a PNI of less than 45 was shown to be the strongest risk factor for the occurrence of complications after hepatectomy in patients with HCC. The mechanism by which a low PNI leads to poor prognosis remains unclear; however, malnutrition and hypoalbuminemia, and lymphopenia due to liver cirrhosis (either singly or in combination) may underlie this process. Conversely, a poor prognosis may be improved by appropriate preoperative nutritional therapy or immunonutrition, as an ample caloric supply and enhanced immune system increase resistance to complications.^36^–^39^ Wang et al. demonstrated that a low PNI was a poor prognostic factor for overall and disease-free survival in patients with HCC.^30^ They showed that a systemic inflammatory response played a significant role in the development and progression of HCC. Hypoalbuminemia reflected the presence of cancer cachexia caused by a sustained inflammatory response, either from the tumor itself or as a host reaction.^40^ Lymphocytes are crucial components of the immune system and play important roles in biological processes including cancer initiation, proliferation, differentiation, and metastasis.^41^,^42^ Therefore, our finding that the PNI is an independent risk predictor for postoperative complications suggests the feasibility of its use as a preoperative assessment tool for patients with HCC.

In this study, we included much earlier surgical cases to acquire the necessary number of cases. Therefore, we also analyzed only cases in the last 10 years (*n* = 392) and observed similar results (data not shown). We aimed to use preoperative factors alone to predict postoperative complications in this paper. The assumption is that some degree of prediction can be easily made when a patient with HCC visits at the outpatient clinic. When surgical factors were included in addition to preoperative factors, ASA, PNI, and intraoperative blood transfusion were identified as predictive factors (data not shown).

Recent studies have demonstrated that compared to the use of major hepatectomy, the use of minimally invasive hepatectomy for HCC, including laparoscopic surgery, was associated with significantly lower postoperative mortality and complication rates, and shorter hospitalizations.^43^,^44^ These results indicate that patients require substantial nutritional and immunological support after undergoing massive hepatectomy due to the excessive invasive stress they sustain in such surgeries. Compared to patients with high PNI, those with low PNI may have poorer nutritional and immunological healing ability. Furthermore, liver cirrhosis leads to poor liver function and regeneration capacity in patients. Thus, it is expected that patients with low PNI would have higher rates of postoperative complications than patients with high PNI.

Several limitations are associated with the current study. First, the retrospective nature of this study had a relatively small number of patients who underwent major hepatectomy. Further prospective studies are needed to confirm our conclusions. Second, this study included all patients who underwent hepatectomy, regardless of the type of surgical procedure. The low-PNI group had high rates of partial resection; therefore, the results of this study may be biased. Third, the mortality rate of 2.9% was worse than that currently reported. Twelve of 15 patients who died after surgery underwent major hepatectomies including right trisegmentectomy and central bisegmentectomy, and extended right lobectomy, most of which were performed before 2010. Additionally, intraoperative blood loss was significantly higher in patients who died postoperatively in the complication group (mean blood loss in patients who died after surgery vs surviving patients, 2870 mL vs 1415 mL, *P* = 0.007).

In conclusion, we have demonstrated that a preoperative PNI of less than 45 was the most powerful predictor of complications after hepatic resection among patients with HCC. When encountering patients with HCC who have low PNI values in the outpatient clinic, an appropriate method of treatment (reduction surgery or interventional treatment) should be considered or preoperative nutrition should be provided until the day of surgery.

## References

[1] El-Serag HB, Rudolph KL (2007). Hepatocellular carcinoma: epidemiology and molecular carcinogenesis. Gastroenterology..

[2] Grazi GL, Ercolani G, Pierangeli F (2001). Improved results of liver resection for hepatocellular carcinoma on cirrhosis give the procedure added value. Ann Surg..

[3] Taketomi A, Kitagawa D, Itoh S (2007). Trends in morbidity and mortality after hepatic resection for hepatocellular carcinoma: an institute’s experience with 625 patients. J Am Coll Surg..

[4] Zaydfudim VM, Vachharajani N, Klintmalm GB (2016). Liver resection and transplantation for patients with hepatocellular carcinoma beyond Milan criteria. Ann Surg..

[5] Tustumi F, Ernani L, Coelho FF (2018). Preoperative strategies to improve resectability for hepatocellular carcinoma: a systematic review and meta-analysis. HPB (Oxford)..

[6] Okamura Y, Takeda S, Fujii T (2011). Prognostic significance of postoperative complications after hepatectomy for hepatocellular carcinoma. J Surg Oncol..

[7] Harimoto N, Shirabe K, Ikegami T (2015). Postoperative complications are predictive of poor prognosis in hepatocellular carcinoma. J Surg Res..

[8] Ruan DY, Lin ZX, Li Y (2015). Poor oncologic outcomes of hepatocellular carcinoma patients with intra-abdominal infection after hepatectomy. World J Gastroenterol..

[9] Yang T, Liu K, Liu CF (2019). Impact of postoperative infective complications on long-term survival after liver resection for hepatocellular carcinoma. Br J Surg..

[10] Shimada M, Takenaka K, Fujiwara Y (1998). Risk factors linked to postoperative morbidity in patients with hepatocellular carcinoma. Br J Surg..

[11] Belghiti J, Hiramatsu K, Benoist S (2000). Seven hundred forty-seven hepatectomies in the 1990s: an update to evaluate the actual risk of liver resection. J Am Coll Surg..

[12] Yamashita Y, Hamatsu T, Rikimaru T (2001). Bile leakage after hepatic resection. Ann Surg..

[13] Virani S, Michaelson JS, Hutter MM (2007). Morbidity and mortality after liver resection: results of the patient safety in surgery study. J Am Coll Surg..

[14] Sadamori T, Yagi S, Shinoura Y (2013). Risk factors for major morbidity after liver resection for hepatocellular carcinoma. Br J Surg..

[15] Kenjo A, Miyata H, Gotho M (2014). Risk stratification of 7732 hepatectomy cases in 2011, from the National Clinical Database for Japan. J Am Coll Surg..

[16] Gibbs J, Cull W, Henderson W, Daley J, Hur K, Khuri SF (1999). Preoperative serum albumin level as a predictor of operative mortality and morbidity: results from the National VA Surgical Risk Study. Arch Surg..

[17] Lien YC, Hsieh CC, Wu YC (2004). Preoperative serum albumin level is a prognostic indicator for adenocarcinoma of the gastric cardia. J Gastrointest Surg..

[18] Ray-Coquard I, Cropet C, Van Glabbeke M (2009). European Organization for Research and Treatment of Cancer Soft Tissue and Bone Sarcoma Group. Lymphopenia as a prognostic factor for overall survival in advanced carcinomas, sarcomas, and lymphomas. Cancer Res..

[19] Schwegler I, von Holzen A, Gutzwiller JP, Schlumpf R, Mühlebach S, Stanga Z (2010). Nutritional risk is a clinical predictor of postoperative mortality and morbidity in surgery for colorectal cancer. Br J Surg..

[20] Szkandera J, Pichler M, Absenger G (2014). The elevated preoperative platelet to lymphocyte ratio predicts decreased time to recurrence in colon cancer patients. Am J Surg..

[21] Okugawa Y, Toiyama Y (2019). Yamamoto A, et al.

[22] Onodera T, Goseki N, Kosaki G (1984). Prognostic nutritional index in gastrointestinal surgery of malnourished cancer patients. Nippon Geka Gakkai Zasshi.

[23] Nozoe T, Ninomiya M, Maeda T, Matsukuma A, Nakashima H, Ezaki T (2010). Prognostic nutritional index: A tool to predict the biological aggressiveness of gastric carcinoma. Surgery Today..

[24] Kanda M, Fujii T, Kodera Y, Nagai S, Takeda S, Nakao A (2011). Nutritional predictors of postoperative outcome in pancreatic cancer. Br J Surg..

[25] Watanabe M, Iwatsuki M, Iwagami S, Ishimoto T, Baba Y, Baba H (2012). Prognostic nutritional index predicts outcomes of gastrectomy in the elderly. World J Surg..

[26] Mohri Y, Inoue Y, Tanaka K, Hiro J, Uchida K, Kusunoki M (2013). Prognostic nutritional index predicts postoperative outcome in colorectal cancer. World J Surg..

[27] Sun K, Chen S, Xu J, Li G, He Y (2014). The prognostic significance of the prognostic nutritional index in cancer. a systematic review and meta-analysis. J Cancer Res Clin Oncol..

[28] Peng W, Li C, Wen TF (2016). Postoperative prognostic nutrition index change is an independent predictor of survival in patients with small hepatocellular carcinoma. Am J Surg..

[29] Ke M, Xu T, Li N (2016). Prognostic nutritional index predicts short-term outcomes after liver resection for hepatocellular carcinoma within the Milan criteria. Oncotarget..

[30] Wang Z, Wang J, Wang P (2018). The prognostic value of prognostic nutritional index in hepatocellular carcinoma patients: a metaanalysis of observational studies. PLoS One..

[31] Shimada M, Takenaka K, Gion T (1996). Prognosis of recurrent hepatocellular carcinoma: A 10-year surgical experience in Japan. Gastroenterology..

[32] Barbosa-Silva MC (2008). Subjective and objective nutritional assessment methods: what do they really assess?. Curr Opin Clin Nutr Metab Care..

[33] Rungsakulkij N, Vassanasiri W, Tangtawee P (2019). Preoperative serum albumin is associated with intra-abdominal infection following major hepatectomy. J Hepatobiliary Pancreat Sci..

[34] Poulia KA, Klek S, Doundoulakis I (2017). The two most popular malnutrition screening tools in the light of the new ESPEN consensus definition of the diagnostic criteria for malnutrition. Clin Nutr..

[35] Cederholm T, Jensen GL, Correia MITD (2019). GLIM Core Leadership Committee, GLIM Working Group. GLIM criteria for the diagnosis of malnutrition – A consensus report from the global clinical nutrition community. J Cachexia Sarcopenia Muscle..

[36] Yao H, Bian X, Mao L, Zi X, Yan X, Qiu Y (2015). Preoperative enteral nutritional support in patients undergoing hepatectomy for hepatocellular carcinoma: A strengthening the reporting of observational studies in epidemiology artible. Medicine..

[37] Thornblade LW, Varghese TK, Shi X (2017). Preoperative immunonutrition and elective colorectal resection outcome. Dis Colon Rectum..

[38] Gianotti L, Besselink MG, Sandini M (2018). Nutritional support and therapy in pancreatic surgery: A position paper of the international study group on pancreatic surgery (ISGPS). Surgery..

[39] Adiamah A, Skorepa P, Weimann A, Lobo DN (2019). The impact of preoperative immune modulating nutrition on outcomes in patients undergoing surgery for gastrointestinal cancer: A systematic review and meta-analysis. Ann Surg..

[40] McMillan DC (2008). An inflammation-based prognostic score and its role in the nutrition-based management of patients with cancer. The Proceedings of the Nutrition Society..

[41] Unitt E, Marshall A, Gelson W (2006). Tumor lymphocytic infiltrate and recurrence of hepatocellular caricinoma following liver transplantation. J Hepatol.

[42] Mano Y, Aishima S, Fujita N (2013). Tumor-associated macrophage promotes tumor progression via STAT3 signaling in hepatocellular carcinoma. Pathobiology..

[43] Takahara T, Wakabayashi G, Beppu T (2015). Long-term and perioperative outcomes of laparoscopic versus open liver resection for hepatocellular carcinoma with propensity score matching: a multi-institutional Japanese study. J Hepatobiliary Pancreat Sci..

[44] Andreou A, Struecker B, Raschzok N (2018). Minimal-invasive versus open hepatectomy for hepatocellular carcinoma: Comparison of postoperative outcomes and long-term survivals using propensity score matching analysis. Surg Oncol..

